# The Effect of Direct-to-Plant Styrene-Butadiene-Styrene Block Copolymer Components on Bitumen Modification

**DOI:** 10.3390/polym11010140

**Published:** 2019-01-15

**Authors:** Wengang Zhang, Zhirong Jia, Yixia Zhang, Kui Hu, Longting Ding, Fang Wang

**Affiliations:** 1 School of Civil and Architectural Engineering, Shandong University of Technology, Zibo 255049, China; ziwuzizwg@sdut.edu.cn; 2 School of Engineering and Information Technology, The University of New South Wales, Australian Defence Force Academy, Canberra, ACT 2600, Australia; Y.zhang@adfa.edu.au; 3 School of Computing, Engineering and Mathematics, Western Sydney University, Penrith, NSW 2751, Australia; 4 College of civil engineering and architecture, Henan University of Technology, Zhengzhou 450001, China; mailhukui@163.com; 5 School of Highway, Chang’an University, Xi’an 710064, China; dltphd2018@163.com; 6 School of Physics and Optoelectronic Engineering, Shandong University of Technology, Zibo 255049, China; wangfangsdut@163.com

**Keywords:** direct-to-plant SBS, EVA, naphthenic oil, fluorescence microscopy, binary picture, melting point index, bitumen

## Abstract

Five types of material, styrene-butadiene-styrene block copolymer (SBS), ethyl-vinyl-acetate (EVA), naphthenic oil, maleic anhydride grafted ethylene-vinyl acetate copolymer (EVA-*g*-MAH) and butylated hydroxytoluene (BHT) were used as the raw ingredients for manufacturing direct-to-plant SBS in this paper. Thirteen kinds of direct-to-plant SBS with different EVA/SBS and naphthenic oil/SBS were prepared as well as the processes diagrammatic sketch of dispersion and swelling of direct-to-plant SBS modifier in bitumen were discussed. Microscopic images of direct-to-plant SBS modified bitumen with different components were obtained using fluorescence microscopy. The micro-images were analysed and quantified with MATLAB software. The influence of key components on the micro-morphology of direct-to-plant SBS-modified bitumen is discussed, followed with the tests on melting points and the melting indexes of direct-to-plant SBS with different EVA/SBS and naphthenic oil/SBS. The performances test of bitumen and bituminous mixture modified by these direct-to-plant SBS were also conducted. Results show that, with the ratio improvement of EVA/SBS or naphthenic oil/SBS, the number of the pixel dot number of area (SBS) in microscopic images increased. Enlargement of the pixel dot number of centre line elongate and the structure fineness was observed, indicating that the dispersion and swelling effect of the SBS modifier in bitumen had been improved. Meanwhile, the macro index, such as the melting point and melting index of direct-to-plant SBS, was also improved corresponding to the increase of EVA/SBS ratio or naphthenic oil/SBS ratio. With the addition of EVA or naphthene oil content, penetration and ductility of direct-to-plant SBS modified bitumen received gradual enhancement, but the softening point and viscosity were found out to be decreased. The high-temperature and low-temperature performances of direct-to-plant SBS modified bituminous mixture can be effectively improved by adding EVA or naphthenic oil. By meeting the required performances of direct-to-plant SBS, modified bitumen and bituminous mixture, the component of direct-to-plant SBS is recommended as, SBS:EVA:naphthenic oil:EVA-*g*-MAH:BHT is 1:0.1–0.5:0.05–0.2:0.03:0.05. For the compatibleness of SBS with different bitumen are different, necessary tests verification is recommended to be carried out prior to usage.

## 1. Introduction

Styrene-butadiene-styrene block copolymer (SBS)-modified bitumen is the most widely used modified bitumen worldwide [1,2]. Factory manufacturing is the main production process [3,4,5]. SBS-modified bitumen is produced by fully grinding, dispersing and swelling with a shearing machine or a colloid mill [6,7,8,9,10]. However, separation will occur easily due to the density difference between SBS and bitumen in the transportation or the storage [11]. In addition, the SBS modifier has thermal decomposition problem in bitumen; that is, molecule breakage and degradation would occur under a long-term high-temperature condition, resulting in the continuous decline of softening point and ductility as well as the declining of the performances of SBS modified bitumen after it is produced [12,13,14]. Segregation and thermal decomposition seriously reduce the performance of SBS-modified bitumen. In order to solve the problems, Guolu Gaoke Engineering Technology Institute Co., Ltd. (Beijing, China) [15] put forward the concept of direct-to-plant SBS, i.e., SBS modified bituminous mixture can be directly prepared by adding a direct-to-plant SBS modifier in the mixing process, and its performances are similar to the common SBS modified bituminous mixture. This required that the direct-to-plant SBS modifier must have a higher solubility and a faster adsorption swelling capacity in bitumen. In 2018, “Technical Guideline for Construction of Direct-to-Plant SBS Modified Bituminous Pavement (T/CHTS 20003-2018)” [15] was promulgated in China. This specification provided guidance for the application of direct-to-plant SBS, modified bitumen and bituminous mixture.

Through the analysis and research on a large amount of literature, the key principle of direct-to-plant SBS was that SBS and other substances were premixed to reduce its melting point and increase its solution rate in bitumen. It had been reported that ethyl-vinyl-acetate (EVA) had good flexibility and elasticity, which can be dissolved in bitumen [16]. The mixing of EVA and SBS could improve the solubility of SBS in bitumen, and it could also improve the high-temperature and low temperature-performance of modified bitumen [17]. Naphthenic oil, from petroleum, has a saturated cyclic carbon chain [18,19], is miscible with SBS and highly compatible with bitumen [20,21]. Compatibilizer, which is the main component was aromatherapy, was necessary to improve the solubility of SBS in bitumen. In addition, butylated hydroxytoluene (BHT) would play an important role in reducing thermal decomposition and improving oxidation resistance of SBS [22,23]. Although it had been reported that direct-to-plant SBS modifiers could be prepared by using SBS, EVA, naphthenic oil, compatibilizer and antioxidant, but the main problems were yet to be solved. They are:

(1) Without shearing, the dispersion and swelling process of direct-to-plant SBS modifier in bitumen is still unknown.

(2) The effect of components proportion (especially EVA/SBS and naphthenic oil/SBS) on the micro-morphology of direct-to-plant SBS modified bitumen are still unknown.

(3) No reports have been made regarding the effect of components proportion (especially EVA/SBS and naphthenic oil/SBS) on the macro index of direct-to-plant SBS modified bitumen.

(4) There is no scientific basis for the proportion range of each component in the existing direct-to-plant SBS modifier.

Therefore, it is of importance to investigate the proportion of each component in a direct-to-plant SBS modifier from both micro and macro perspectives. In recent years, the development of microimaging has provided support for microresearch, especially the invention of the fluorescence microscope [24,25]. When the short wavelength light of fluorescence microscope is used to irradiate SBS modified bitumen film, the SBS emits yellow light, while bitumen and other substances are basically black [26,27,28]. With the help of the fluorescence microscope, it is possible to observe and analyze the dispersing and swelling process of direct-to-plant SBS modifier in bitumen. MATLAB software has powerful image processing functions, especially in binary image processing and skeleton image processing so that the quantitative calculation of the dispersed swelling degree of SBS can be realized [29]. For the macro index, some researchers had used the melting index as an important evaluation indicator of direct-to-plant SBS modifier [30]. It had been reported that the higher the melt index, the better the dispersibility of direct-to-plant SBS modifier in asphalt [15]. It was also reported that the lower the melting point, the faster the melting rate of direct-to-plant SBS modifier in bitumen [15,31,32]. In addition, the technical performances of direct-to-plant SBS modified bituminous mixture should not be worse than that of the common bituminous mixture [33]. These research results have an important reference value for this paper.

In this paper, SBS, EVA, naphthenic oil, maleic anhydride grafted ethylene-vinyl acetate copolymer (EVA-*g*-MAH) and BHT were used as the raw ingredients of the direct-to-plant SBS. Many kinds of direct-to-plant SBS with different EVA/SBS and naphthenic oil/SBS were prepared. The process diagrammatic sketch of dispersion and swelling of direct-to-plant SBS modifier in bitumen were given in this paper. Microscopic images of direct-to-plant SBS modified bitumen with different components were obtained by fluorescence microscopy. With the help of MATLAB software, the microimages were analysed and quantified. The influence of key components on the micro-morphology of direct-to-plant SBS modified bitumen was discussed. The melting points and the melting indexes of direct-to-plant SBS with different EVA/SBS and naphthenic oil/SBS were tested. The performances of bitumen and bituminous mixture with these direct-to-plant SBS were also tested. On the basis of test data, the composition range of direct-to-plant SBS is recommended.

## 2. Raw Materials

### 2.1. Raw Materials of Direct-to-Plant SBS

In this paper, the direct-to-plant SBS modifier consisted of SBS, EVA, naphthenic oil, EVA-*g*-MAH and BHT. SBS and EVA were produced by Sinopec Yanshan Petrochemical Company (Beijing, China), the particle size of SBS was less than 0.0374 mm and the melting index of EVA was 18.5 g/10min. Naphthenic oil was produced by Miosonio Co., Ltd (Huizhou, China). EVA-*g*-MAH was produced by Zhenjiang Daoyi Material Technology Co., Ltd (Zhenjiang, China), and the BHT was produced by Chinasun Specialty Products Co., Ltd (Changshu, China).

### 2.2. Preparation Process of Direct-to-Plant SBS

**Figure 1:**
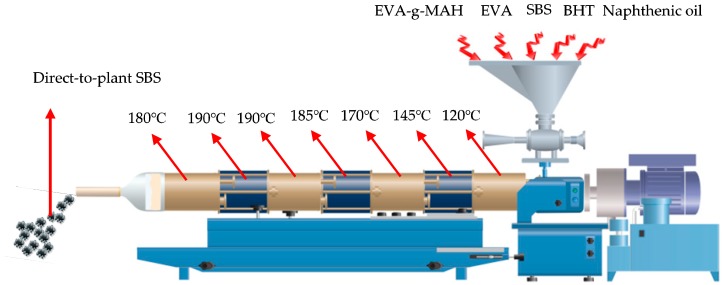
The preparation process of direct-to-plant SBS modifier.

A certain proportion of SBS, EVA, EVA-*g*-MAH and BHT was mixed evenly, then put into the a double screw extruder for melting and granulation. The double screw extruder was set into seven zones by the temperature, and the temperature from the feeding zone to the wire drawing zone was controlled at 120, 145, 170, 185, 190, 190 and 180 °C, respectively, the precision was within ±2 °C, the particle size of direct-to-plant SBS was less than 2 mm, as shown in Figure 1 below. 

**Figure 2:**
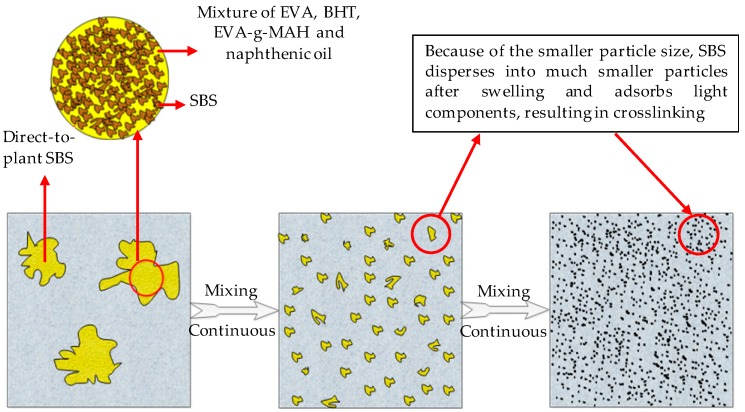
Diagrammatic sketch of the dispersion and swelling process of direct-to-plant SBS modifier in bitumen.

The principle of rapid melting of the direct-to-plant SBS modifier in bitumen is described as follows: The SBS modifier with smaller particle size is melt-blended with EVA beforehand, and EVA acts as a solvent and better disperses SBS. The solubility parameters of EVA and bitumen are similar, which make the mixture of SBS and EVA disperse quickly and evenly after being put into bitumen. Due to the small particle size, the swelling rate of SBS is quite fast and the degree of swelling is relatively high. In addition, naphthenic oil and EVA-*g*-MAH have very large solubility, which helps SBS to disperse uniformly in EVA, and also improve the swelling capacity of SBS in bitumen greatly. Figure 2 is the diagrammatic sketch of the dispersion and swelling process of direct-to-plant SBS modifier in bitumen.

### 2.3. Preparation Process of Modified Bitumen by Direct-to-Plant SBS

A certain quality of direct-to-plant SBS was put into bitumen at the temperature of 180 °C, then the direct-to-plant SBS modified bitumen could be prepared without shearing by continuous stirring for 1 h. Compared with ordinary SBS modified bitumen, direct-to-plant SBS-modified bitumen had no complicated shearing process and shorter swelling time.

### 2.4. Bitumen, Aggregate and Mineral Powder

**Figure 3:**
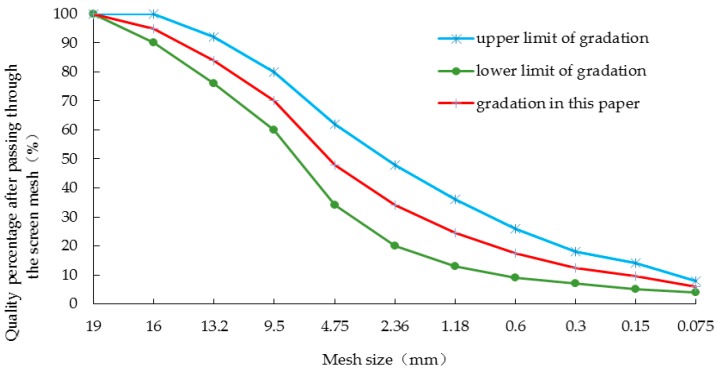
The grading curve of AC-16.

A-90 bitumen [33] used in this paper was produced by Shandong Chambroad Petrochemicals Co., Ltd. (Binzhou, China). The technical indicators were shown in Table 1. The aggregate was basalt, the mineral powder was produced by limestone, and the technical indicators were shown in Table 2. The gradation type used in this paper was a dense-graded bituminous mixture with a nominal maximum particle size 16 mm (AC-16). The grading curve designed according to the specification is shown in Figure 3 [33].

### 2.5. Preparation Process of the Modified Bituminous Mixture by Direct-to-Plant SBS

First, a certain quality of direct-to-plant SBS was mixed with hot aggregate for 60 s; Second, a certain quality of bitumen was mixed for 90 s; then, the mineral powder was mixed for 90s. Lastly, the modified bituminous mixture was put into an oven at 163 ± 2 °C for 2 h.

## 3. Test Design

Guolu Gaoke Engineering Technology Institute Co. Ltd. (Beijing, China) has done a lot of research on direct-to-plant SBS modifiers. Research results show that EVA can improve the compatibility between direct-to-plant SBS modifier and bitumen by virtue of its similar dissolution parameters with bitumen, whereas naphthene oil has a certain solubility to SBS, which can improve the dispersion and swelling speed of SBS in bitumen. At the same time, EVA, naphthenic oil and SBS do not react with each other during the preparation of modifier, which makes it possible to study the effects of EVA/SBS or naphthenic oil/SBS on the properties of direct-to-plant SBS modifier [15]. According to the previous research [21], the ratio of SBS to compatibilizer and BHT has a relatively optimal value, i.e., SBS:compatibilizer:BHT = 1:0.03:0.05. Therefore, in this paper, the relationship between SBS, EVA and naphthenic oil was focused on. For convenience, the dosage of SBS was fixed to 1 in this paper. Table 3 below gives the different kinds of direct-to-plant SBS.

The direct-to-plant SBS modifiers with different components was prepared according to the method given in Section 2.2. Then, the key macro index of the direct-to-plant SBS modifier, including melting point and melting index, were tested. The results helped to reveal the relationship between SBS and EVA or naphthenic oil. Then the direct-to-plant SBS modified bitumen was prepared according to the method shown in Section 2.3, where the content of modifier was controlled using SBS (4.5%, bitumen mass percentage). Then the microimages of direct-to-plant SBS-modified bitumen with different components were obtained using fluorescence microscopy (Shanghai Optical Instrument Factory, Shanghai, China). The microimages were analysed and quantified by using MATLAB software. Then the performances of the direct-to-plant SBS modified bitumen samples were tested, such as penetration, ductility, softening point, etc. Modified bituminous mixture using direct-to-plant SBS was prepared according to the method shown in Section 2.5, where the bitumen aggregate ratio of the modified bituminous mixture by direct-to-plant SBS was 4.8%. Then, the performances were tested based on “Standard Test Methods of Bitumen and Bituminous mixtures for Highway Engineering JTG E20-2011” [35], such as dynamic stability (DS), residual stability (MS’), tensile strength ratio (TSR) and maximum bending tensile strain ($\varepsilon_{B}$).

## 4. Microstructure Analysis

### 4.1. Micro-Images Processing Method of Direct-to-Plant SBS Modified Bitumen

When the SBS-modified bitumen film is irradiated by the short wavelength light comes from fluorescence microscope, the SBS emits yellow light while bitumen and other substances are basically black, such that the interface between SBS and bitumen is clear. In this paper, the fluorescence microscope for modified bitumen (model number: WMX-3930), produced by Shanghai Optical Instrument Factory (Shanghai, China) was used to achieve the microscopic images of direct-to-plant SBS modified bitumen, i.e. red-green-blue images (RGB). The test process was described as follows: The prepared direct-to-plant SBS modified bitumen was dripped onto the slide glass, the cover glass was gently pushed from one side to the other, the bubbles were removed during the pushing process, and the thickness should be uniform. Then the RGB of the direct-to-plant SBS-modified bitumen could be achieved using the WMX-3930 under the condition of 500× magnification. Each RGB was a series of pixels sorted in a particular order, i.e. pixel matrix. Pixels at each location stored a set of colour codes, as shown in Equation (1).

(1) $$F=\left(\begin{array}{ccc} f\left(1,1\right) & \cdots & f\left(1,N\right)\\ \vdots & \ddots & \vdots \\ f\left(M,1\right) & \cdots & f\left(M,N\right) \end{array}\right)$$

where *F* is pixel matrix function, *M* is the number of rows, and *N* is the number of columns.

RGB is stored as a *M* × *N* × 3 pixel data matrix in MATLAB, where the colour of each pixel is determined by the intensity combination of red (R), green (G) and blue (B). If each colour is represented by a numerical value between 0 to 1, the three colours of each pixel are stored in the third dimension of the array, such as (0,0,0) for black and (1,1,1) for white. The binary image is the image of the pixel value that takes a value of 0 or 1: 0 is black, 1 is white and there is no intermediate grey value. By using the “im2bw” function in MATLAB and selecting threshold parameters in level [0,1], RGB images can be transformed into binary images. In binary images, SBS appeared as a white colour, while other substances appeared as a black colour. The “bwarea” function can be used to read the pixel dot number of the white area. When the content of SBS is the same, the bigger the number of the pixel dot number of the white area, the better the swelling effect of SBS. By using the “bwmorph” function in MATLAB, binary images can be skeletonized, i.e. the centre line of the white area can be found. The “bwarea” function can also be used to read the pixel dot number of the centre line. When the content of SBS is the same, the bigger the number of the pixel dot number of the centre line, the better the crosslinking effect of SBS. The fineness of SBS structure can be calculated by dividing the pixel dot number of the white area by the pixel dot number of the centre line. When the content of SBS in bitumen is the same, the thicker the fineness of SBS structure, the higher the strength of SBS structure.

In this paper, the processing methods and commands used in image processing is shown as follows.

All RGB images are set to 254 × 192 pixels.
Command: A = imread(‘C:\Users\z3525296\Desktop\1\9#.jpg’)%;
Set level = 0.55;
Command: B = im2bw(A, 0.55)%;
Selected ‘skel’ morphological operation and ‘Inf’ (Infinite) operation.
Command: C = bwmorph(B, ‘skel’, Inf)%;
Command: X = bwarea(A)%
Command: Y = bwarea(B)%
Command: Z = x/y%

where A is the RGB, B is the binary image, C is the skeletonized image, X is the pixel dot number of white area (SBS), Y is the pixel dot number of the centre line and Z is the fineness of SBS structure.

**Figure 4:**
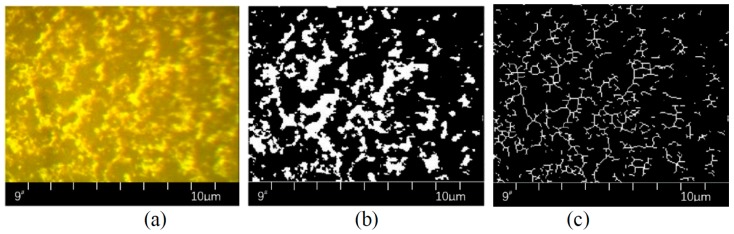
Image processing of: (**a**) RGB, (**b**) binary image and (**c**) skeletonized image.

Figure 4 below is an example.

### 4.2. Micro-Images of Direct-to-Plant SBS Modified Bitumen

**Figure 5:**
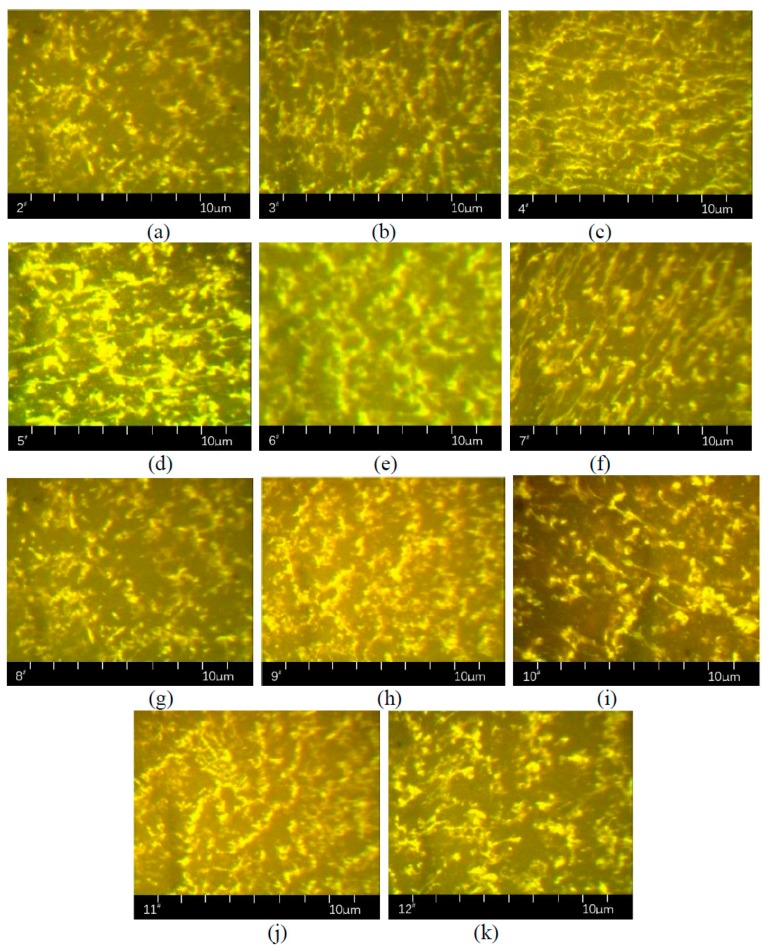
The representative RGB of direct-to-plant SBS modified bitumen modifier: (**a**) 2^#^, (**b**) 3^#^, (**c**) 4^#^, (**d**) 5^#^, (**e**) 6^#^, (**f**) 7^#^, (**j**) 8^#^, (**h**) 9^#^, (**i**) 10^#^, (**j**) 11^#^ and (**k**) 12^#^.

The direct-to-plant SBS modified bitumen was prepared according to Table 3, the content of modifier was controlled using SBS (4.5%, bitumen mass percentage). In order to reduce the error, the sample number of each modified bitumen should not be less than 10. The modified bitumen using 1^#^ and 13^#^ modifiers mentioned in Table 3 had particulates with incomplete melting, meaning the RGB acquisition failed. The representative RGB of the other kinds of modified bitumen are shown in Figure 5 below.

The yellow area in Figure 5 is the SBS modifier, and the black area is bitumen or other substances. Except for 1^#^ and 13^#^, the 2^#^–12^#^ direct-to-plant SBS modifier prepared in this paper dispersed at the micron level in bitumen, and the swelling and crosslinking have occurred. However, the degree of swelling and crosslinking are different between different direct-to-plant SBS modifiers. Taking 5^#^ and 8^#^ as examples, in these two pictures, the area of SBS modifier and the structure fineness were quite different.

**Figure 6:**
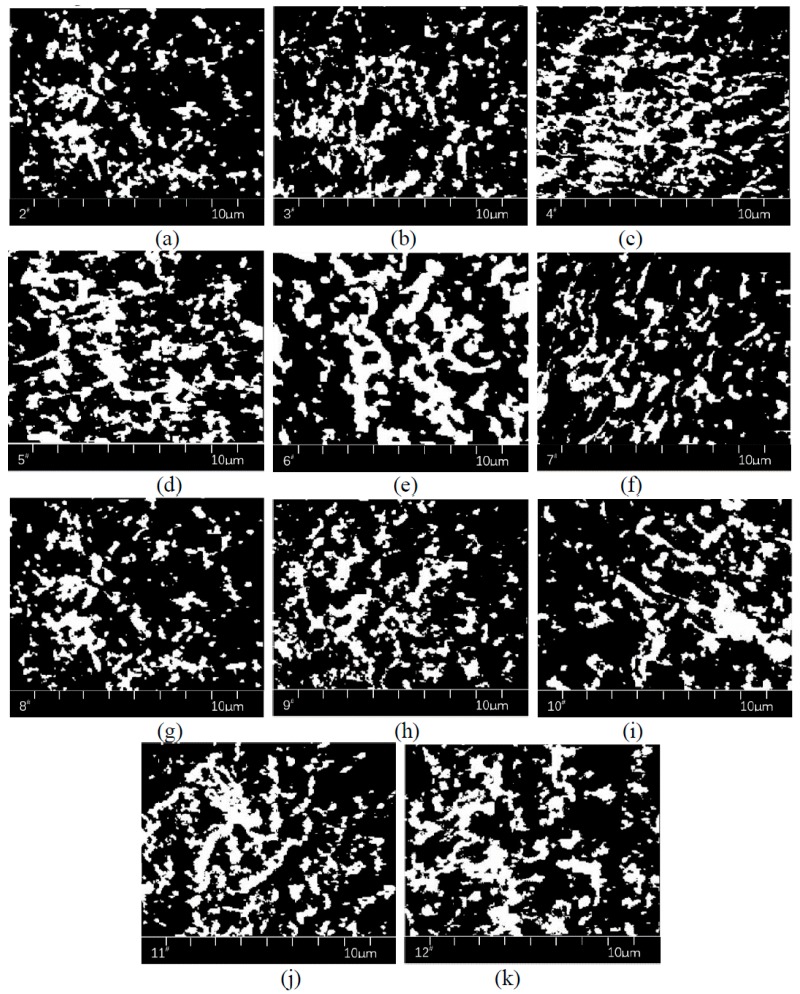
The representative binary image of direct-to-plant SBS modified bitumen modifier: (**a**) 2^#^, (**b**) 3^#^, (**c**) 4^#^, (**d**) 5^#^, (**e**) 6^#^, (**f**) 7^#^, (**j**) 8^#^, (**h**) 9^#^, (**i**) 10^#^, (**j**) 11^#^ and (**k**) 12^#^.

**Figure 7:**
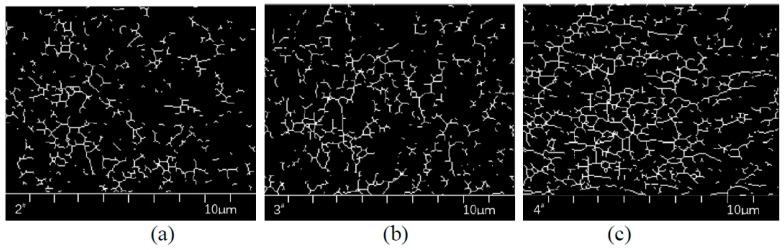

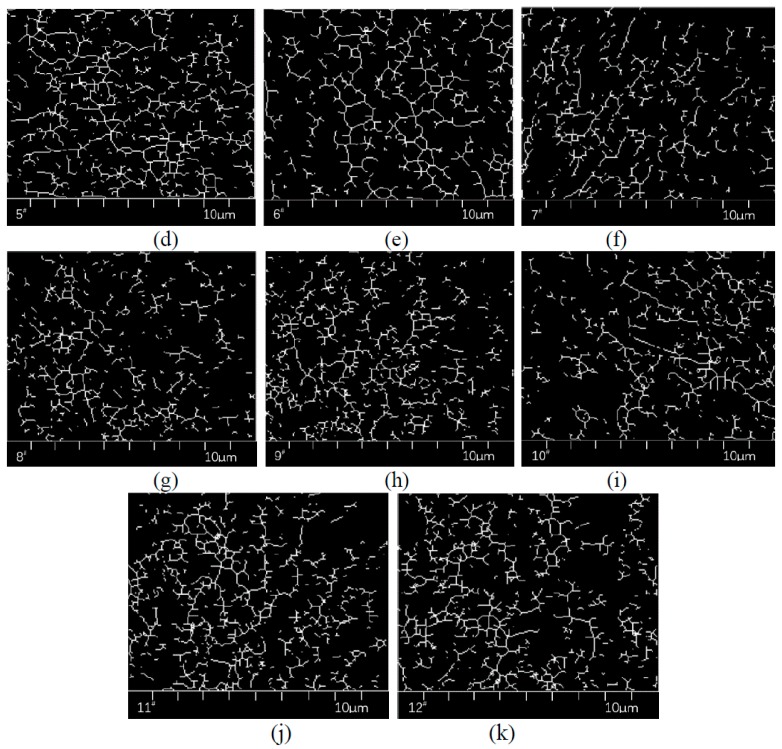
The representative skeletonized image of direct-to-plant SBS modified bitumen modifier: (**a**) 2^#^, (**b**) 3^#^, (**c**) 4^#^, (**d**) 5^#^, (**e**) 6^#^, (**f**) 7^#^, (**j**) 8^#^, (**h**) 9^#^, (**i**) 10^#^, (**j**) 11^#^ and (**k**) 12^#^.

The area of SBS in Figure 5 was quantitatively analyzed using binary processing, as shown in Figure 6. In order to analyze the fineness of the SBS structure after swelling, the skeletonized image as shown in Figure 7 was obtained via the skeletonization of Figure 6.

### 4.3. Analysis and Calculation on Microscopic Images of Direct-to-Plant SBS Modified Bitumen

In this paper, with the help of the “bwarea” function, the pixel dot number of the white area and the pixel dot number of the centre line was calculated, then the fineness of the SBS structure was calculated. In order to reduce the errors, 10 RGB images were selected for each direct-to-plant SBS modified bitumen to be processed and calculated. The average results are summarized in Table 4 below.

**Figure 8:**
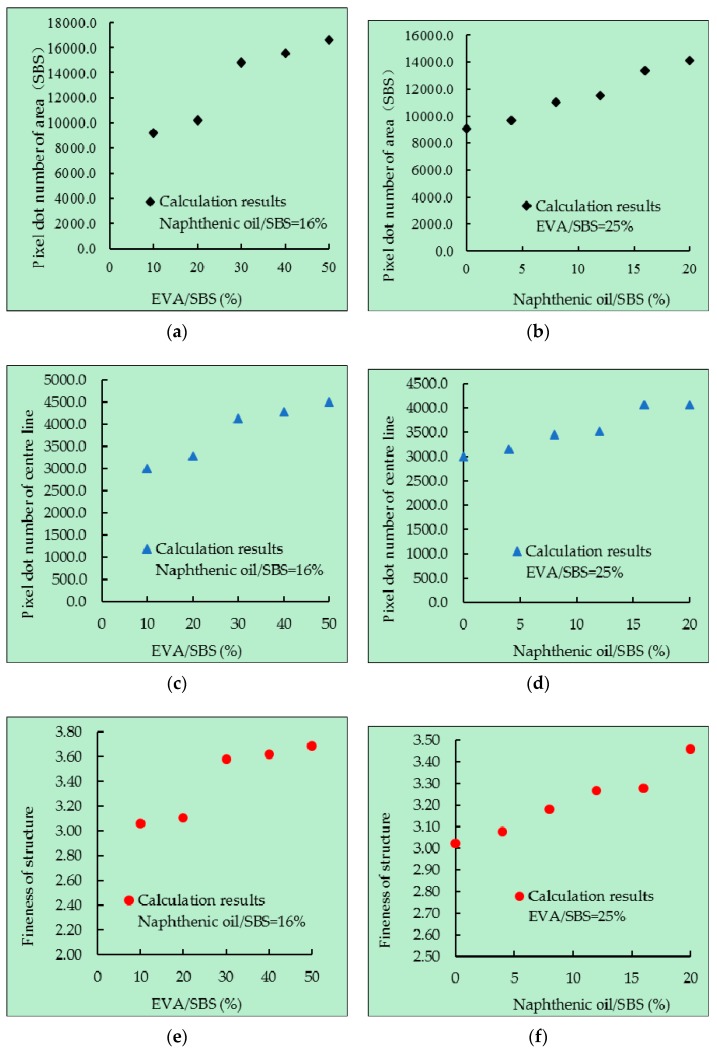
The relation between components of direct-to-plant SBS and microstructure parameters, when naphthenic oil/SBS = 16%: (**a**) pixel dot number of area (SBS), (**c**) pixel dot number of centre line (SBS) and (**e**) fineness of structure. When EVA/SBS = 25%: (**b**) pixel dot number of area (SBS), (**d**) pixel dot number of centre line (SBS) and (**f**) fineness of structure.

Table 4 shows that the microstructural parameters of different direct-to-plant SBS modified bitumen were different. The larger the pixel dot number of area (SBS), the larger the pixel dot number of the centre line, the thicker the SBS structure fineness and the better the dispersion and swelling effect of SBS. Figure 8 shows the relationship among EVA/SBS, naphthenic oil/SBS, pixel dot number of area (SBS), pixel dot number of the centre line and the structure fineness of SBS.

As can be seen from Figure 8, the pixel dot number of area (SBS), the pixel dot number of the centre line and the structure fineness of SBS all increased linearly with the increase of EVA/SBS, which indicates that the proper increase of the proportion of EVA contributed to the dispersion and melting of SBS particles in bitumen. The principle is described as follow: During the preparation of direct-to-plant SBS modifier, with the increase of EVA/SBS, the SBS particles were better dispersed in the modifier. Because of the similar solubility parameters with bitumen, EVA could dissolve rapidly in the process of mixing with bitumen, which made the SBS particles disperse rapidly and evenly in bitumen. In addition, the quite small size of SBS particles was also an important factor for its rapid swelling.

As can also be seen from Figure 8, the pixel dot number of area (SBS), the pixel dot number of the centre line and the structure fineness of SBS all increased linearly with the increase of naphthenic oil/SBS, which indicates that the proper increase of the proportion of naphthenic oil contributed to the dispersion and melting of SBS particles in bitumen. The principle is described as follows: Naphthene oil itself is a subsidiary product of petroleum, which is highly compatible with bitumen, with a saturated hydrocarbon content of 87.55%–93.86% and an aromatic hydrocarbon content of 6.14%–11.96%. It is similar to light components of bitumen and has a certain degree of mutual solubility with SBS. Therefore, during the preparation of direct-to-plant SBS modifiers, SBS had begun to swell, which undoubtedly shortened the swelling process of SBS in bitumen. In addition, naphthenic oil also improved the solubility of SBS in bitumen.

From the microscopic point of view, the higher the content of EVA and naphthenic oil, the better the dispersion and melting effect of direct-to-plant SBS modifier in bitumen. In order to guarantee the macro-technical performance of direct-to-plant SBS modified bitumen, “Technical Guideline for Construction of Direct-to-Plant SBS Modified Bituminous Pavement (T/CHTS 20003-2018)” stipulates that the content of SBS in a direct-to-plant SBS modifier should not be less than 50% [15]. Relatively high economic costs also limit the content of EVA and naphthenic oil in direct-to-plant SBS modifiers.

## 5. Macroscopic Test Results and Analysis

Although the micro-analysis can observe and analyze the advantages and disadvantages of the components of direct-to-plant SBS modifier, the macro-index was still used as the control index in the standards. Therefore, the macro-performance indexes of different components of direct-to-plant SBS modifier, modified bitumen and bituminous mixtures were tested and analysed in this paper.

### 5.1. Melting Point

**Figure 9:**
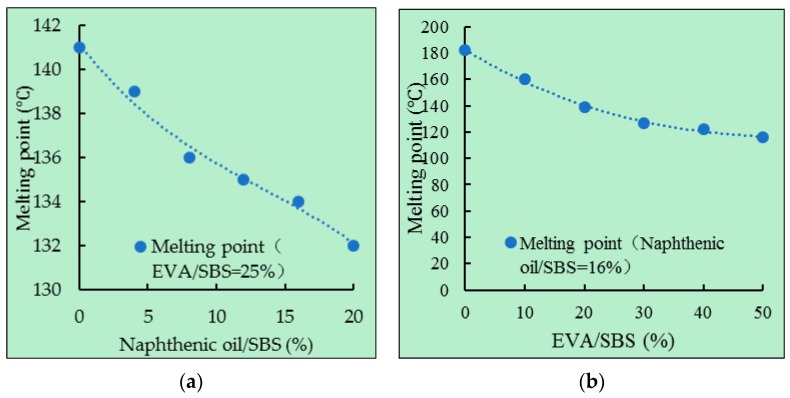
Melting points of different direct-to-plant SBS modifier: (**a**) EVA/SBS = 25% and (**b**) naphthenic oil/SBS = 16%.

The melting points of direct-to-plant SBS modifier with different components were tested. The results are shown in Figure 9.

As can be seen from Figure 9, when naphthenic oil/SBS was 16%, the melting point of direct-to-plant SBS reduced with the increase of EVA/SBS. When EVA/SBS was 25%, the melting point of direct-to-plant SBS reduced with the increase of naphthenic oil/SBS. This was mainly due to the melting point of EVA being 75 °C, and naphthenic oil is liquid at room temperature. With the admixture of EVA or naphthenic oil into SBS, the melting point of the mixture was bound to decrease.

### 5.2. Melting Index

**Figure 10:**
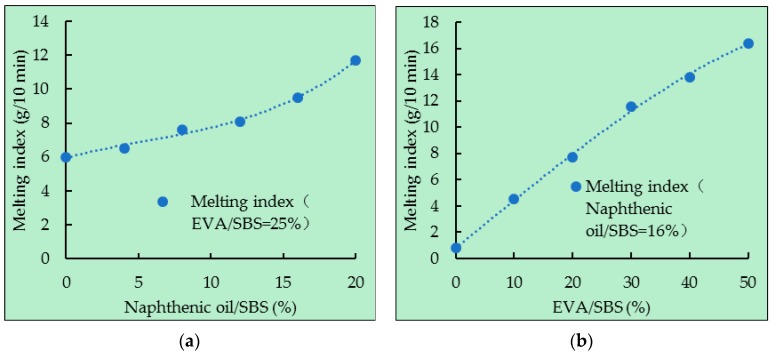
Melting index of different direct-to-plant SBS modifiers: (a) EVA/SBS = 25% and (b) naphthenic oil/SBS = 16%.

The melting index is the mass of direct-to-plant SBS solution passing through the standard capillary tube within 10 minutes at 190 °C and 21.2 N pressure. Its unit is g/10 min. The melting index of direct-to-plant SBS modifiers with the different component are shown in Figure 10.

As can be seen from Figure 10, the melting index of direct-to-plant SBS increased with the increase of EVA/SBS or naphthenic oil/SBS. There are two reasons, the first one is that the viscosity of EVA and naphthenic oil at 190 °C are very small, which can reduce the viscosity of the mixture. Another reason is that naphthenic oil can dissolve SBS, which leads to an effective melting of SBS and EVA. According to the Technical Guideline for Construction of Direct-to-Plant SBS Modified Bituminous Pavement (T/CHTS 20003-2018) [15], the melting index of direct-to-plant SBS cannot be less than 2.0 g/10 min. If naphthenic oil/SBS was 16%, when EVA/SBS < 3.3%, the melting index broke the rule.

### 5.3. Performances of Modified Bitumen Using Direct-to-Plant SBS

Modified bitumen using different direct-to-plant SBS were prepared according to the method shown in Section 2.3. The test results are shown in Table 5.

There are certain differences in the performances of modified bitumen using different direct-to-plant SBSs. The worst performance occurred when there was no EVA, and naphthenic oil in the modifier followed without EVA. The penetration, ductility and the properties after RTFOT will increase with the increase of EVA/SBS or naphthenic oil/SBS, while at the same time, viscosity and the softening point will reduce. It was remarkable that when EVA/SBS was 0% and 10%, there were some incomplete melted modifier granules. This indicates that EVA was the key component of direct-to-plant SBS preparation.

### 5.4. Performances of the Modified Bituminous Mixture Using Direct-to-Plant SBS

By using the modified bituminous in Table 5, modified bituminous mixtures using direct-to-plant SBS were prepared under the method shown in Section 2.4. The gradation type was AC-16, and the bitumen aggregate ratio was 4.8%. The test results are shown in Table 6.

As can be seen from Table 6, when there was no EVA and naphthenic oil in the modifier, all performances of the modified bituminous mixtures were the worst. DS and ε_B_ increased with the increase of EVA/SBS or naphthenic oil/SBS, and the change of EVA/SBS had a greater impact on these two performances. At the same time, the water stability was slightly improved.

## 6. The Range of the Key Component

The test results show that, as the key component of direct-to-plant SBS preparation, EVA played an important role in the performances of bitumen and bituminous mixtures modified using direct-to-plant SBS. When the naphthenic oil/SBS was 16%, the performances of modified bitumen and bituminous mixtures using direct-to-plant SBS met the requirements of the specifications (the Ministry of Communications Highway Science Institute, JTG E20-2011 [35]), while EVA/SBS was in the range of 0.1–0.5%. Table 4 below is the permissible proportion range of EVA when naphthenic oil/SBS is 16%.

**Figure 11:**
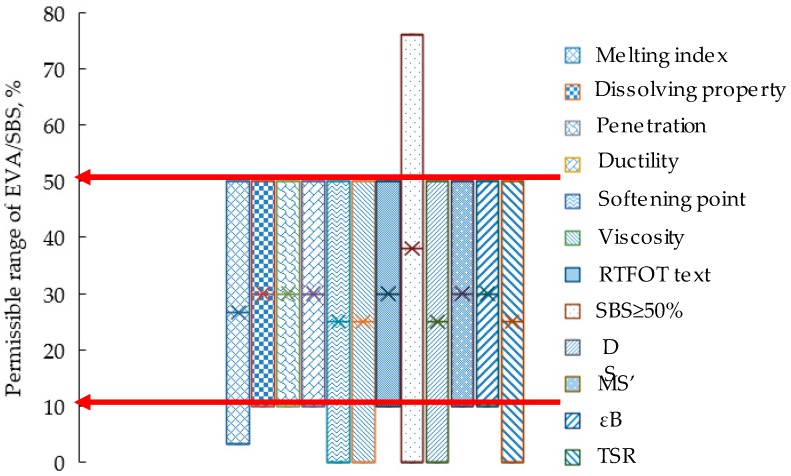
The permissible proportion range of EVA when naphthenic oil/SBS =16%.

Each bar in Figure 11 represents a limiting factor (technical properties of direct-to-plant SBS modifier, technical properties of modified bitumen and the performances of modified bituminous mixture), and the ordinate is the range of EVA/SBS. For example, for MS’, the requirements in the relevant specification were not less than 85% [33], according to the data in Table 6; when EVA/SBS was between 10% and 50%, it met the requirement. Similarly, when other pavement performances of direct-to-plant SBS-modified asphalt mixtures meet the specification, the corresponding range of EVA/SBS could be calculated according to Table 6. The range of EVA/SBS corresponding to the technical properties of direct-to-plant SBS modified bitumen could be calculated according to the data in Table 5 [33]. The technical requirements of the direct-to-plant SBS modifier itself mainly refers to the melting index (≥2.0 g/10 min) and the phenomenon of mixing (no particle residue) [28], where the range of EVA/SBS can be determined using Figure 10 and related experimental phenomena.

From the microscopic point of view, the higher the content of EVA and naphthenic oil, the better the dispersion and melting effect of the direct-to-plant SBS modifier in bitumen. When EVA/SBS of direct-to-plant SBS modifier was more than 0.1, it could achieve a better dispersion and melting effect in bitumen. In addition, analysis from the perspective of the melting index, dissolution in bitumen, SBS proportion, penetration, ductility, softening point, viscosity and properties after RTFOT, DS, TSR, MS’ and εB, the permissible proportion range of EVA when naphthenic oil/SBS =16% is 0.1–0.5. What needs to be explained is that when naphthenic oil/SBS ≠ 16%, the possibility of small-range data floating should not be ruled out. Based on the above research, the components of direct-to-plant SBS recommended in this paper is SBS:EVA:naphthenic oil:EVA-*g*-MAH:BHT = 1:0.1–0.5:0.05–0.2:0.03:0.05. It is suggested that necessary test verification should be carried out before use.

## 7. Conclusions

Direct-to-plant SBS can be prepared by mixing SBS, EVA, naphthenic oil, EVA-*g*-MAH and BHT. It can be directly mixed with bitumen and aggregate to prepare the bituminous mixture, which reduces the preparation of SBS modified bitumen. The main conclusions in this paper can be draw as follows:

(1) With the EVA/SBS or the naphthenic oil/SBS increases, the dispersion and swelling effect of the SBS modifiers in bitumen are improved. As the number of the pixel dot number of area (SBS) in the microimage of direct-to-plant SBS modified bitumen increased, the pixel dot number of centre line will elongated, as well as the enlargement of the structure fineness of SBS. 

(2) With the EVA/SBS or naphthenic oil/SBS increase, the performances of direct-to-plant SBS modifier, modified bitumen and the modified bituminous mixture improved. 

(3) Under the premise of meeting all specifications, when EVA/SBS = 25%, the permissible proportion range of naphthenic oil/SBS is 5–20%, the permissible proportion range of EVA when naphthenic oil/SBS = 16% is 10%–50%. The components of direct-to-plant SBS recommended in this paper is SBS:EVA:naphthenic oil:EVA-*g*-MAH:BHT = 1:0.1–0.5:0.05–0.2:0.03:0.05.

## Figures and Tables

**Table 1:** A-90 bitumen technical indicators.

Test	Unit	Measured Value	Test Items	Unit	Measured Value
60 °C Dynamic viscosity	Pa.s	204.7	Asphaltene	%	11.1
10 °C Ductility	cm	56.9	Saturate	%	18.3
15 °C Ductility	cm	>100	Aromatic	%	40.7
15 °C Density	g/cm^3^	0.997	Resin	%	29.9
Softening point	°C	48.6	25 °C Penetration	0.1mm	88.4
RTFOT	Penetration ratio			%	69.8
	Loss of quality			%	0.7
	10 °C Residue Ductility			cm	19
	15 °C Residue Ductility			cm	23

RTFOT: Rotary film oven test.

**Table 2:** Technical indicators of aggregate and filler.

Aggregate and Filler	Technical Indicators	Measured Value	Technical Requirement [33]			Test Methods [34]
			Expressway and Class-I Highway		Other Classified Highway	
			Surface Layer	Other Layer		
**Coarse aggregate**	Crushed stone value (%)	22.4	≤26	≤28	≤30	T0316
	Worn stone vale (%)	13.6	≤28	≤30	≤35	T0317
	Water absorption (%)	0.9	≤2.0	≤3.0	≤3.0	T0304
	Flat and elongated particle (%)	4	≤15	≤18	≤20	T0314
**Fine aggregate**	Sand equivalent value (%)	72	≥60	≥60	≥50	T0334
	Angularity (%)	49	≥30	≥30	----	T0345
	Methylene blue value (g/kg)	23.7	≤25	≤25	----	T0346
	Sediment percentage (%)	1.2	≤3	≤3	≤5	T0333
**Mineral filler**	Hydrophilic coefficient	0.89	≤1	≤1	≤1	T0353
	Plasticity index	3.1	≤4	≤4	≤4	T0354
	Moisture content (%)	0.5	≤1	≤1	≤1	T0103
	Apparent density (t/m^3^)	2.712	≥2.50	≥2.50	≥2.45	T0352

**Table 3:** The different kinds of direct-to-plant SBS modifier.

No. of Modifier	SBS	EVA	Naphthenic Oil	Compatibilizer	BHT
1^#^	1	0	0.16	0.03	0.05
2^#^	1	0.1	0.16	0.03	0.05
3^#^	1	0.2	0.16	0.03	0.05
4^#^	1	0.3	0.16	0.03	0.05
5^#^	1	0.4	0.16	0.03	0.05
6^#^	1	0.5	0.16	0.03	0.05
7^#^	1	0.25	0	0.03	0.05
8^#^	1	0.25	0.04	0.03	0.05
9^#^	1	0.25	0.08	0.03	0.05
10^#^	1	0.25	0.12	0.03	0.05
11^#^	1	0.25	0.16	0.03	0.05
12^#^	1	0.25	0.2	0.03	0.05
13^#^	1	0	0	0.03	0.05

**Table 4:** Computing results of different kinds of direct-to-plant SBS modified bitumen.

Types of Modifiers	Pixel Dot Number of the White Area (SBS)	Pixel Sot Number of the Centre Line	Fineness of SBS Structure
1^#^	--	--	--
2^#^	9230.4	3013.7	3.06
3^#^	10205.0	3286.3	3.11
4^#^	14802.0	4132.8	3.58
5^#^	15536.0	4285.3	3.63
6^#^	16642.0	4510.4	3.69
7^#^	9068.6	2998.1	3.02
8^#^	9688.9	3148.5	3.08
9^#^	11013.0	3460.8	3.18
10^#^	11514.0	3524.5	3.27
11^#^	13342.0	4069.6	3.28
12^#^	14111.0	4079.1	3.46
13^#^	--	--	--

**Table 5:** Performances of modified bitumen by direct-to-plant SBS.

No.		1^#^	2^#^	3^#^	4^#^	5^#^	6^#^	7^#^	8^#^	9^#^	10^#^	11^#^	12^#^	13^#^	Technical Requirement of SBSI-C [33]
**Penetration (25 °C, 0.1 mm)**		57.6	62.7	66.3	67.9	69.5	72.1	61.8	65.3	65.7	66.1	66.5	67.4	89.6	60–80
**Softening point (°C)**		81.0	74.8	66.7	57.1	49.6	43.9	66.7	64.2	63.3	63.1	62.6	62.3	47.9	≥55
**Ductility (5 °C, cm)**		29.4	37.5	39.1	39.6	41.4	42.7	37.8	37.0	39.6	40.2	39.7	40.7	22.6	≥30
**Viscosity (135 °C, Pa·s)**		1.777	1.703	1.614	1.497	1.374	1.291	1.566	1.571	1.586	1.591	1.608	1.647	1.103	≤3
**RTFOT**	**Penetration ratio (%)**	84.3	79.6	78.3	80.7	82	79.9	80.2	78.2	80.1	80.4	79.6	79.4	68.7	≥60
	**Mass loss (%)**	0.05	0.1	0.1	0.2	0.2	0.2	0.1	0.1	0.3	0.2	0.2	0.2	0.8	≤1.0
	**Residual ductility (5 °C, cm)**	18.3	23.8	24.1	26.5	27.3	29.8	24.1	25.7	25.6	26.1	26.8	27.4	12.3	≥20
**Completely dissolved (Y/N)**		N	Basic	Y	Y	Y	Y	Basic	Y	Y	Y	Y	Y	N	----

Y: Completely dissolved; N: Not completely dissolved; Basic: Basic dissolved.

**Table 6:** Performances of modified bituminous mixtures by direct-to-plant SBS.

No.	1^#^	2^#^	3^#^	4^#^	5^#^	6^#^	7^#^	8^#^	9^#^	10^#^	11^#^	12^#^	13^#^	Performance Requirement [33]
**VV (%)**	3.5	3.6	3.5	3.5	3.5	3.4	3.5	3.5	3.6	3.5	3.4	3.5	3.4	3–5
**MS’ (%)**	81	92	95	93	93	95	95	92	93	94	93	92	83	85
**TSR (%)**	78	93	89	93	92	90	86	90	92	94	95	94	76	80
**DS (times/mm)**	4658	6836	7133	7268	7336	7405	6875	7152	6937	7436	7250	7309	4403	2400
**ε_B_, με**	2439	3469	3521	3617	3664	3702	3550	3558	3628	3589	3607	3676	2319	3000

VV: void volume, MS’: residual stability, TSR: tensile strength ratio, DS: dynamic stability,.$\varepsilon_{B}$: maximum bending tensile strain; Performance requirement: The highest standard specified in the related specification [33].
